# Evaluation of community-based interventions to improve TB case detection in a rural district of Tanzania

**DOI:** 10.9745/GHSP-D-14-00026

**Published:** 2014-05-13

**Authors:** Charlotte Colvin, Jackson Mugyabuso, Godwin Munuo, John Lyimo, Eyal Oren, Zahra Mkomwa, Mohammed Makame, Atuswege Mwangomale, Vishnu Mahamba, Lisa Mueller, D'Arcy Richardson

**Affiliations:** a U.S. Agency for International Development, Washington, DC, USA; b PATH Tanzania, Dar es Salaam, Tanzania; c University of Arizona, Mel and Enid Zuckerman College of Public Health, Tucson, AZ, USA; d PATH, Washington, DC, USA; e Independent Consultant, San Francisco, CA, USA

## Abstract

In Tanzania, people with tuberculosis (TB) commonly self-medicate or visit traditional healers before seeking formal medical care. Between 2009 and 2011, we piloted a community-based project in Kisarawe District to improve TB case notification. The project trained 15 traditional healers and 15 pharmacists to identify and refer individuals with TB symptoms to diagnostic facilities. In addition, the project trained 2 community members to collect and fix sputum from symptomatic individuals onto slides, which they then delivered by bicycle to the nearest diagnostic facility. To determine effectiveness, we analyzed routine case detection data and referrals from traditional healers and pharmacists and conducted a cross-sectional survey of recently diagnosed smear-positive TB patients (N = 150) to understand their treatment-seeking behavior. From 2009 to 2011, smear-positive TB case notification increased by 68% in Kisarawe District, from 28/100,000 to 47/100,000, even while TB case notification nationally stayed the same (at approximately 14/100,000). The traditional healers and pharmacists referred 434 people with presumptive TB to diagnostic facilities, 419 of whom (97%) went to the facilities; of those who went to facilities for testing, 104 people (25%) were diagnosed with TB. The percentage of new TB case notifications that were referred through the network ranged from 38% to 70% per reporting quarter. Sputum fixers collected and delivered specimens from 178 individuals, 17 of whom (10%) were diagnosed with TB. Almost 60% of surveyed smear-positive TB patients first visited a pharmacist or traditional healer before seeking care at a diagnostic facility. These results prompted scale up of community interventions to 9 more districts in 2011 and to another 26 districts in 2013. Establishing referral networks that bring TB information and services closer to community members can contribute to improved TB case notification.

## Abstract (precis)

Enlisting traditional healers and pharmacists to improve TB detection contributed 38% to 70% of new smear-positive case notifications per quarter in a rural district of Tanzania.

## INTRODUCTION

In 2006, the World Health Organization (WHO) and the Stop TB Partnership launched the “Stop TB Strategy”^1^ and published “The Global Plan to Stop TB, 2006–2015.”^2^ Both are key strategy documents aimed at broadening the scope of tuberculosis (TB) program implementation to include efforts needed to achieve global TB targets, which include the Millennium Development Goal of halting and reversing the incidence of TB by 2015. In 2012, an estimated 8.6 million people around the world developed TB, and 1.3 million people died from the disease.^3^

Recognizing that the TB prevention, care, and treatment strategies used up to the time the strategy documents were published would not be sufficient to reach the 2015 target, the WHO strategy included empowering people with TB and affected communities as a central component, with the aim of supporting comprehensive, community-based responses.^1^ Community participation in disease prevention, diagnosis, care, and treatment has been recognized as a critical element in recent documents and initiatives.^4^^,^^5^

Community-based approaches have been recognized as critical to preventing and treating TB.

The WHO “Global Tuberculosis Report 2013” noted that Tanzania has reached a treatment success rate of 88% among smear-positive cases and a smear-positive case detection rate of 79%.^3^ Despite these laudable accomplishments, TB and TB/HIV co-infection continue to pose a substantial burden on the health system in Tanzania and remain a significant cause of morbidity and mortality; almost 40% of all TB patients with known HIV status are co-infected with HIV.^3^ Similar to other high-burden countries for TB, Tanzania's National Tuberculosis and Leprosy Programme (NTLP) is investing in training, new diagnostic technologies, implementation of TB/HIV collaborative activities, infection prevention and control, community-based approaches, and other activities to improve case detection and to build on their accomplishments in treatment success.

Despite the emphasis placed on community-based approaches globally and within the NTLP strategy, there are few published data on the effectiveness of these interventions on TB outcomes. With support from the U.S. Agency for International Development (USAID) and in collaboration with the NTLP, PATH designed and implemented a package of innovative community-based activities to improve TB case notification in Kisarawe District in Pwani Region. From 2009 to 2011, PATH's district TB/HIV coordinator worked with local officials to implement, monitor, and evaluate the interventions and to collect data on key outputs and outcomes associated with improved case notification, with the goal of identifying effective and feasible models to scale up throughout Tanzania. The unique efforts undertaken in Kisarawe District provided an opportunity for Tanzania to play a significant role in informing the global TB community about which interventions may yield gains in case detection and how to monitor and evaluate such community-based approaches.

A referral network between community providers and TB diagnostic facilities was established to reduce diagnostic delays.

## INTERVENTION DESCRIPTION

The interventions were piloted and evaluated in Kisarawe District, located in Pwani Region in the Coastal Zone, between 2009 and 2011. Kisarawe is a predominantly rural area, with a population of about 100,000, bordering metropolitan Dar es Salaam. Although recent district-level data are not available, a 2004 health profile for the Coastal Zone estimated that communicable diseases account for 44% of the total burden of disease, including malaria, TB, and HIV/AIDS.^7^ In 2009, the baseline smear-positive case notification rate was 31/100,000, and the TB/HIV co-infection rate among TB cases with known HIV status was 34%.^8^

Efforts to improve TB case notification through community-based approaches included:

- **Sensitizing regional and district TB coordinators, community leaders, and community-based organizations** on the importance of community-based interventions to support the local TB program. Local PATH staff met with stakeholders, including the Council Health Management Team, to obtain their support for improving TB diagnosis and treatment services in general and for the new interventions specifically. Additionally, the PATH team consulted with Community's Own Resource Persons (CORPs) and a community-based organization, MKUKI, formed by former TB patients, to request their support and participation in implementing the interventions.
- **Training pharmacists and traditional healers** to identify and refer individuals with TB symptoms for follow up and further evaluation in public-sector DOTS (Directly Observed Therapy Short-Course) diagnostic facilities. As in other settings, it was assumed that many people with TB symptoms seek care first from a pharmacist or traditional healer before going to a public-sector DOTS facility. To address potential diagnostic delays, PATH introduced a formal referral network between willing pharmacists and traditional healers and the DOTS diagnostic centers. In July 2009, 15 pharmacists and 15 traditional healers received 2-day training on basic information about TB and DOTS-based diagnosis and treatment, proper screening of symptomatic individuals, and how to use referral slips and registers to track referrals to DOTS. The participants also received a directory of DOTS facilities in Kisarawe to facilitate referral of symptomatic individuals.
- **Training,** **deployment, and supervision of 2 sputum fixers—**community members who collected sputum from symptomatic individuals at remote facilities that did not have smear microscopy; prepared and “fixed” slides; and then delivered them by bicycle to the nearest DOTS diagnostic facility and assisted in reporting results back to the facility where the individual first presented. This reduced the travel burden on symptomatic individuals and increased access to smear microscopy. The sputum fixers worked sporadically throughout 2009 and 2010.
- **Training 8 current TB patients** to develop a series of informational materials with and for community members based on “TB Photovoice,” a methodology that combines photography with grassroots social action to increase awareness about TB at the community level and provides insight into the day-to-day life of people living with TB. (For further information, see www.tbphotovoice.org.)

This package of community-based interventions was expected to increase the number of symptomatic individuals seeking care in the public-sector DOTS program and to reduce barriers to diagnostic services, which in turn would lead to an increased number of smear-positive TB cases notified and treated in Kisarawe District, as compared with the pre-intervention period. During the implementation period, the PATH TB/HIV district coordinator supervised the interventions and provided ongoing feedback to the pharmacists, traditional healers, sputum fixers, and the TB patients involved with the photography project.[Fig f04]

**Figure f04:**
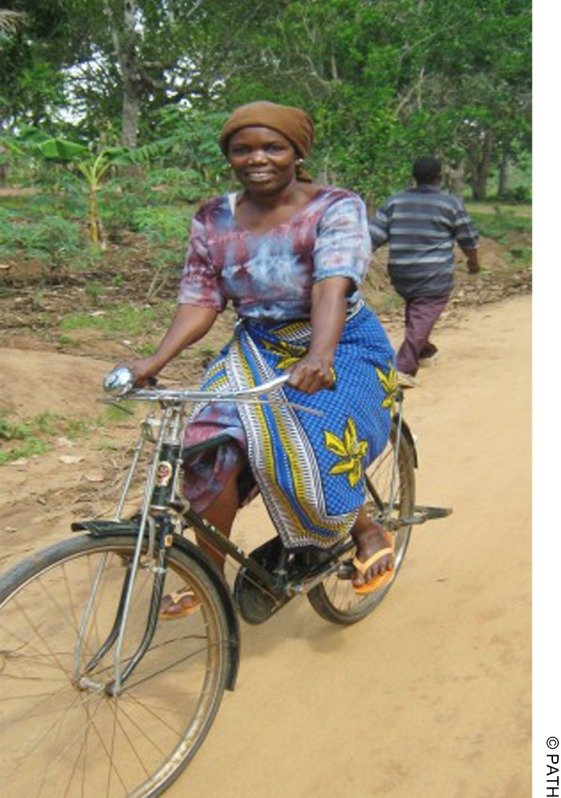
Sputum fixers used bicycles to deliver sputum samples from people with presumptive TB to the nearest diagnostic facility.

## EVALUATION METHODOLOGY

No single data source can address the question of whether a specific intervention or package of interventions results in measurable improvement in TB case notification. Thus, we used a triangulation approach to analyze multiple sources of data that uniquely contribute to our understanding of the effectiveness of community-based approaches.

First, we analyzed routine case notification data from 2009 through 2011 to compare trends prior to implementation, during the start-up phase, and after the interventions were in place for 1 year to assess whether the overall trend in case notification improved over time.

Second, we reviewed programmatic data to determine how well the interventions were performing over time, examining qualitative and quantitative outputs with short- and medium-term outcomes related to case notification. For example, we designed a series of tools for pharmacists and traditional healers to document referrals of symptomatic individuals to DOTS clinics and followed referrals to determine how many were eventually diagnosed with TB in order to estimate the contribution of these activities to overall case notification. We compared programmatic outcomes, such as the number of TB cases diagnosed after referral, to the routine case notification data to determine the contribution of the referral mechanism by quarter.

Finally, we conducted a cross-sectional survey of recently diagnosed smear-positive TB patients (N = 150), using a pretested questionnaire, from October 2010 through March 2011 in Kisarawe District to measure exposure to specific interventions and to assess health-seeking behavior after developing TB symptoms. Approximately half of the interviews took place at the Kisarawe District Hospital, where TB diagnostic procedures take place and treatment is initiated.

These data were entered into SPSS and simple analysis was performed to identify key characteristics of the patients; their knowledge, attitudes, and behavior related to health-seeking for TB symptoms; and their reported experiences with accessing TB diagnostic services. The study received ethical clearance from the PATH Research Ethics Committee based in Seattle, Washington, USA, and from the National Institute for Medical Research in Tanzania.

## RESULTS

### Analysis of Routine Case Detection Data

**FIGURE 1.**
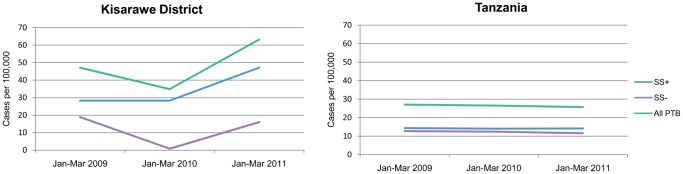
Tuberculosis Case Notification Rates, Kisarawe District, Tanzania, vs. Nationally, 2009–2011 Abbreviations: SS+, sputum smear-positive; SS-, sputum smear-negative; PTB, pulmonary tuberculosis.

From 2009 to 2010, during the pre-intervention and launch period, there was no change in the smear-positive case notification rate (28/100,000). After a full year of implementation (2011), the smear-positive case notification rate increased by 68% to 47/100,000 (Figure 1). The smear-negative case notification rate dropped initially before climbing back to the same level at the start of the intervention.

One year after project implementation, the smear-positive case notification rate increased by 68% in the pilot district.

At the national level, the overall smear-positive case notification rate did not increase during the intervention period (14.3/100,000 in the first quarter of 2009 and 14.1/100,000 during the same reference period in 2011) (Figure 1). Trends for smear-negative and all pulmonary TB cases were similar to the direction observed for smear-positive cases.

### Analysis of Programmatic Data

**FIGURE 2.**
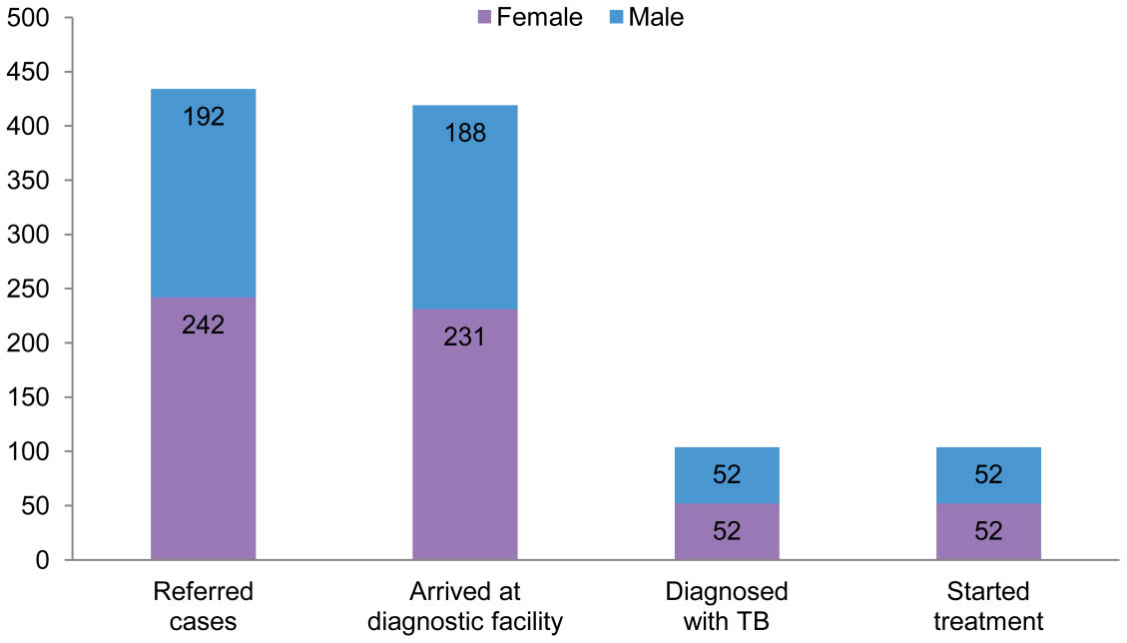
Number of Individuals With Presumptive TB Referred by Pharmacists and Traditional Healers to Diagnostic Facilities, January 2010–March 2011

After pharmacists and traditional healers received training, we followed up with supportive supervision, including ongoing analysis of referrals made by these providers and received at the DOTS facilities. From January 2010 through March 2011, the 30 pharmacists/traditional healers referred 434 individuals with presumptive TB to DOTS facilities, and 419 individuals (97%) arrived at DOTS facilities for further diagnostic testing (Figure 2). Among those who arrived for testing, 104 individuals (25%) were diagnosed with TB (all forms) and started treatment. Among the 30 providers involved in this network, there were clear outliers in terms of their willingness to refer people with symptoms—some pharmacists and traditional healers consistently used the screening tools and referred people who met the criteria for referral each quarter, while others made almost no referrals and did not actively participate in the network.

It is notable that males and females are equally represented in the TB cases that were diagnosed and started treatment after referral, although more females than males were identified and referred by the pharmacists and traditional healers.

**FIGURE 3.**
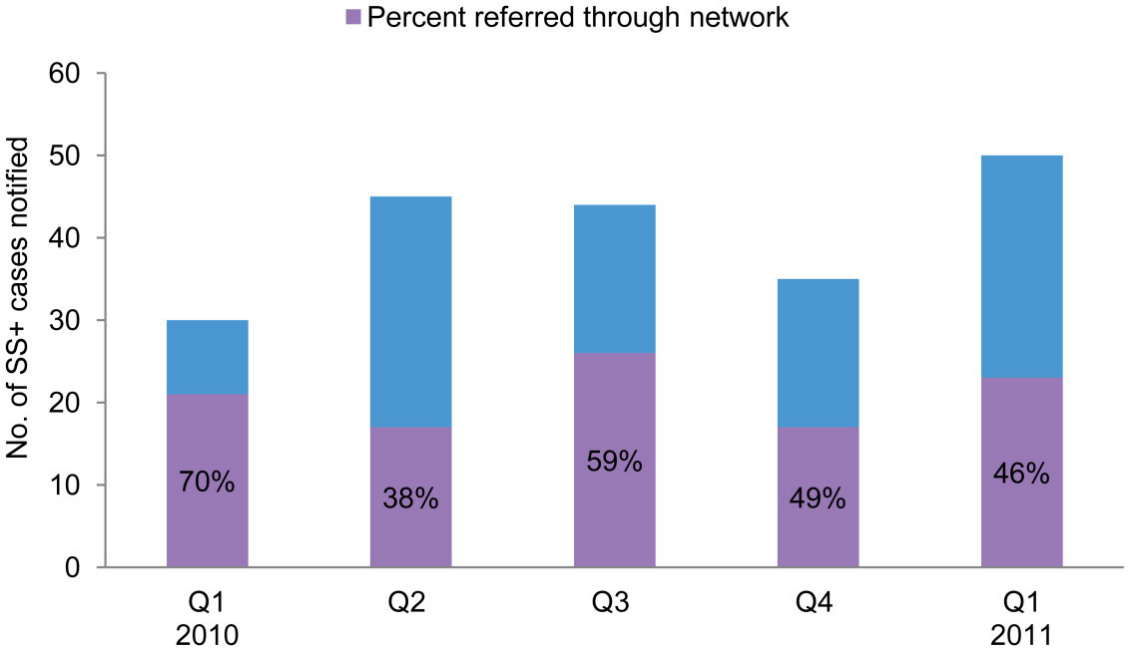
Contribution of Pharmacists and Traditional Healers to Overall TB Case Detection, Kisarawe District, January 2010–March 2011 Abbreviation: SS+, sputum smear-positive.

These data were compared with routine case notification over selected quarters to determine the contribution of the network to overall case notification in Kisarawe. Although we began supervision at the end of 2009, we did not have complete data for assessing the contribution of the intervention to case detection for a full quarter of operation until the first quarter of 2010. The percentage of new TB cases notified that were referred through the network ranged from 38% (in the second quarter of 2010) to 70% (in the first quarter of 2010) (Figure 3).

The referral network contributed 38% to 70% of the new TB case notifications.

Routine output data on the activities of sputum fixers as well as on the photography project were also reviewed to document their potential contribution to case notification. From October 2010 to September 2011, the sputum fixers collected, fixed slides for, and delivered specimens from 178 symptomatic individuals; of these, 17 individuals (10%) were diagnosed with TB and started on treatment.

The resulting photographs and stories from the photography project were used to create educational materials for use in DOTS clinics, private pharmacies, schools, markets, district headquarters, wards, village offices, and in public places throughout the district. Additionally, community leaders used them to educate the community about TB and TB/HIV co-infection.

### Survey of New Smear-Positive TB Patients

All smear-positive TB patients registered for treatment during the survey period were interviewed (N = 150). Two-thirds of the participants were male, and one-third were female. Median age of the respondents was 38 years old, and almost half of respondents were 35–44 years old.

In terms of health-seeking behavior upon developing TB symptoms, almost 60% first visited a pharmacist or traditional healer before seeking care at a public-sector DOTS facility. Additionally, almost half of the respondents visited the DOTS center on the recommendation of a family or household member.

More than two-thirds (70%) of the surveyed smear-positive TB patients mentioned the presence of a CORPs in their village and that CORPs members often led community meetings. The majority (85%) reported that TB was mentioned at the monthly community meetings that they attended. More than 90% also reported seeing information about TB through print media, which are often prominently displayed in the DOTS facilities.

## DISCUSSION

This evaluation of community-based interventions aimed at improving TB case notification in Kisarawe District in Tanzania yielded valuable insights about the potential outcomes of such interventions as well as about the inherent challenges that could compromise optimal program implementation and effectiveness. After 2 years of implementation, the case notification rate for smear-positive TB increased by 68%, and the referral network contributed between 38% and 70% of these notifications.

The survey of new smear-positive TB patients confirmed the importance of pharmacists and traditional healers in the care-seeking pathway of symptomatic individuals, as well as the potential of family and community members such as the CORPs to influence behavior. It also demonstrated the importance of community leaders in educating the public about TB, given the high percentage of survey participants who recalled CORPs' discussion of TB at community meetings. In terms of operationalizing the interventions, the sensitization of district officials and stakeholders was critical for gaining support to conduct the community-based interventions. These findings confirm that NTLP should continue pursuing opportunities to integrate or further exploit the role of traditional healers/pharmacists and community members in the health system. In fact, the positive results of the pilot project prompted scale up to 9 more districts in 2011 and to another 26 districts in 2013.[Fig f05]

**Figure f05:**
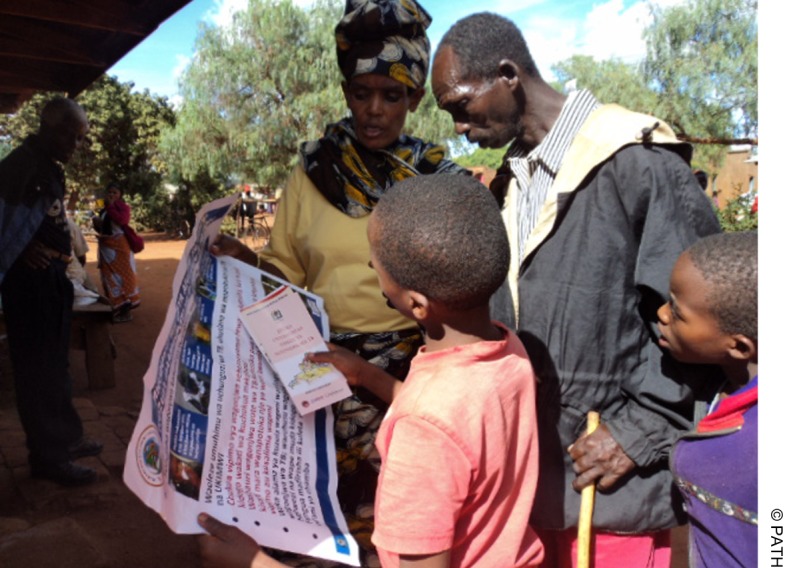
A member of CORPs (Community's Own Resource Persons) shares information about TB with the community.

Pharmacists and traditional healers play an important role in the care-seeking pathway of people with presumptive TB.

At the same time, the evaluation revealed the limitations of community-based approaches in the context of larger health system and other challenges. For example, the initial decrease in smear-negative TB case notifications as the interventions were starting up was likely due to a stockout of X-ray film at Kisarawe Hospital early in 2010, which compromised the ability of the local TB program to confirm and notify smear-negative cases.

Additionally, there are a number of unanswered questions related to “uneven” results, such as the wide variation in the percentage of new TB cases referred through the pharmacist and traditional healer network, which ranged from 38% to 70% per quarter. This could have been due to a real difference in the number of symptomatic people during the first quarter of 2010 (for example, an outbreak of respiratory illness that caused chronic cough but was not TB) or due to changes in referral practice over time. Still, these data show that at the lowest contribution level, almost 40% of TB cases diagnosed during the quarter were referred through the network.

Given variability in the willingness of pharmacists and traditional healers to screen and refer, further investigation of their motivation is needed to effectively expand this intervention. It is worth exploring the performance of the “best” providers in terms of their referral practices and yield of TB cases in order to better understand why they are willing to participate in the intervention and what elements of the model could be refined for improved results. For example, is there a need for additional training or supervision? Are there incentives that might be effective in ensuring participation of the providers? Why were some pharmacists and traditional healers not supportive of the network? Are there certain provider characteristics that may result in better performance? An exploration of these questions may provide valuable insights to inform scale up of the intervention and to ensure that investments are spent wisely on providers who will likely contribute to case detection.

Finally, it is difficult to establish the benefit of training and deploying sputum fixers at the community level to assist with specimen transport to the district laboratory. Although fruitful in terms of supporting TB diagnosis and treatment in remote areas, this intervention was difficult to implement and to supervise. There was no funding to service the bicycles, and the rough geographical terrain limited their use, particularly during the rainy season.

### Limitations

Although this evaluation yielded valuable information about the role of community-based interventions to improve TB diagnosis and treatment, the study design and implementation context have some limitations. First, it was difficult to measure exposure to some interventions in the survey of new smear-positive TB patients. For example, direct measurement of exposure to the informational materials was not captured because they were not specifically “branded” and because there are a number of TB and TB/HIV information, education, and communication materials in use. Second, the intensity of the interventions varied (30 non-DOTS providers trained in referral vs. only 2 sputum fixers), so it is difficult to judge the relative yield of each component without more equal intensity. However, the benefits of including pharmacists and traditional healers in the intervention are clear, given the importance of these providers as a first step along the pathway to TB care and the high rates of referral among some of those trained by the project. Third, due to funding limitations, there was no control district for which we could conduct a similar analysis to compare the different contexts. Future evaluations should include a control arm (to the extent possible) for a more rigorous assessment of the specific interventions. Finally, we do not know how many of the individuals with TB cases notified via referral would have sought care at a DOTS facility in the absence of the referral network, nor do we know whether the referral network decreased the time period between becoming symptomatic and visiting a DOTS diagnostic facility, both issues that should be studied further.

## CONCLUSION

Community-based interventions, such as the establishment of referral networks and other activities that bring TB information and services closer to those with symptoms, can contribute to improved TB case notification. This pilot provides a model for evaluation of community-based approaches to TB case notification that can be applied to similar efforts worldwide. Future research needs include cost-effectiveness analysis to determine the best combination of community-based activities in a given setting.
